# The *TGFB1* Functional Polymorphism rs1800469 and Susceptibility to Atrial Fibrillation in Two Chinese Han Populations

**DOI:** 10.1371/journal.pone.0083033

**Published:** 2013-12-12

**Authors:** Weixing Zheng, Chenghui Yan, Xiaohu Wang, Zhurong Luo, Fengping Chen, Yuhui Yang, Donglin Liu, Xiaobo Gai, Jianping Hou, Mingfang Huang

**Affiliations:** 1 Department of Cardiology, Fuzhou General Hospital, Fujian Medical University, Fuzhou, China; 2 Department of Cardiology, Shenyang General Hospital, Shenyang, China; 3 Department of Cardiology, Fujian Provincial Hospital, Fuzhou, China; Temple University, United States of America

## Abstract

Transforming growth factor-β1 (TGF-β1) is related to the degree of atrial fibrosis and plays critical roles in the induction and perpetuation of atrial fibrillation (AF). To investigate the association of the common promoter polymorphism rs1800469 in the TGF-β1 gene (*TGFB1*) with the risk of AF in Chinese Han population, we carried out a case-control study of two hospital-based independent populations: Southeast Chinese population (581 patients with AF and 723 controls), and Northeast Chinese population (308 AF patients and 292 controls). Two hundred and seventy-eight cases of AF were lone AF and 334 cases of AF were diagnosed as paroxysmal AF. In both populations, AF patients had larger left atrial diameters than the controls did. The rs1800469 genotypes in the *TGFB1* gene were determined by polymerase chain reaction-restriction fragment length polymorphism. The genotype and allele frequencies of rs1800469 were not different between AF patients and controls of the Southeast Chinese population, Northeast Chinese population, and total Study Population. After adjustment for age, sex, hypertension and LAD, there was no association between the rs1800469 polymorphism and the risk of AF under the dominant, recessive and additive genetic models. Similar results were obtained from subanalysis of the lone and paroxymal AF subgroups. Our results do not support the role of the *TGFB1* rs1800469 functional gene variant in the development of AF in the Chinese Han population.

## Introduction

Atrial fibrillation (AF) , the most common clinical arrhythmia, is responsible for substantial morbidity and mortality in the general population [1,2]. AF is associated with changes in cardiac structure and electrical properties known as structural and electrical remodeling. Atrial fibrosis, a hallmark of arrhythmogenic structural remodeling, can lead to increased nonuniform anisotropy in conduction and has been found to be an important substrate for the induction and perpetuation of AF [3–6]. 

In the heart, fibrosis is thought to be partially mediated by transforming growth factor-β1 (TGF-β1), a potent stimulator of collagen-producing cardiomyofibroblasts [3]. There is growing evidence that TGF-β1 can induce atrial fibrosis and play a major role in AF. Using microarray analysis, Barth et al [7] demonstrated upregulated expression of mRNA in the atria of patients with permanent AF. In patients who underwent both open heart surgery for valvular heart disease and the surgical maze procedure for persistent AF, preoperative plasma TGF-β1 levels were related to the degree of atrial fibrosis and could be used to predict the recurrence of AF at the 1-year follow-up after the surgical maze procedure [8]. In patients with non-paroxysmal AF, the plasma TGF-β1 level is an independent predictor of AF recurrence after catheter ablation [9]. In human AF, Gramley et al noted upregulated TGF-β1 expression at the mRNA and protein levels in atrial tissue at different stages of fibrogenesis [10] . 

In canine models, heart failure led to increased atrial TGF-β1 expression and atrial fibrosis, and inhibition of TGF-β1 expression prevented atrial fibrosis and development the AF substrate [11]. In a transgenic mouse model over-expressing constitutively active TGF-β1, there was selective atrial interstitial fibrosis, while ventricular histology was normal [12–14]. This increase in atrial fibrosis was shown to correspond to an increase in conduction heterogeneity and AF vulnerability [13,14]. In another study by the same group, the drug pirfenidone was used to target TGF-β1 expression [15]. Similar results were observed in an experimental model of heart failure, from which increased TGF-β1 expression and atrial fibrosis were reported [11,15], and pirfenidone treatment resulted in a significant reduction of TGF-β1 expression and atrial fibrosis. This reduction in atrial fibrosis also corresponded to a decrease in conduction abnormalities and in AF vulnerability [15]. 

A number of studies have attempted to determine whether naturally occurring single-nucleotide polymorphisms (SNPs) in the TGF-β1 gene (*TGFB1*; gene map locus: chromosome 19q13.1-13.3) affect *TGFB1* expression and TGF-β1 production. For example, A C-to-T SNP at position -509 relative to the first major transcription start site (-509C>T SNP; rs1800469) was found to be differentially related with transcription factor binding to the *TGFB1* promoter, transcriptional activity of *TGFB1*, and TGF-β1 plasma concentration [16,17]. The 868T>C SNP (rs1982073), which gives rise to an amino acid substitution at position 10 (Leu10Pro), was reported to influence steady-state concentrations of *TGFB1* mRNA in peripheral blood mononuclear cells and serum TGF-β1 levels [18,19]. Other SNPs, such as variants at positions -800 (rs1800468), codon 25 (rs1800471, Arg25Pro), and codon 263 (rs1800472, Thr263Ile), were also reported to be functional and can affect TGF-β1 production and/or activation[16,18–23]. It is reasonable to believe that one of these functional genetic variants is associated with the risk of AF. To the best of our knowledge, none of these SNPs has ever been studied in relation to the risk of AF in a general population.

We hypothesized that functional genetic variations in *TGFB1* might contribute to the susceptibility to AF. A high degree of linkage disequilibrium has been observed between rs1800469 and rs1982073 in the Chinese Han population [24–26], whereas the rs1800468, rs1800471, and rs1800472 are extremely rare in the unrelated Chinese individuals [24,26–28]. We focused on rs1800469 as a tagging SNP, to explore its relationship with the risk of AF in two Chinese Han populations in a case-control study.

## Patients and Methods

### Ethics Statement

The Ethics Committees of Fuzhou General Hospital and Shenyang General Hospital approved this study. All subjects provided informed written consent in accordance with the Declaration of Helsinki.

### Populations

The subjects in this study were unrelated Chinese Han from Southeast and Northeast Chinese populations. Subjects from the Southeast Chinese population were patients from Fuzhou General Hospital. We enrolled 581 patients with AF and 723 controls consecutively from June 2007 to April 2010. Subjects from the Northeast Chinese population were patients from Shenyang General Hospital. We enrolled 308 AF patients and 292 controls consecutively from December 2008 to April 2010. 

### Inclusion and Exclusion Criteria

For cases, i.e., AF patients, the inclusion criteria were signs and symptoms of AF confirmed by a cardiologist based on at least one 12-lead resting electrocardiogram (ECG). Controls were enrolled from the same ward during the same period as the cases and verified as being free of AF by a cardiologist, based on ECG or medical files. The controls were individually matched with cases according to the following criteria: sex, age (±5 years), area of residence (from the same province), and presence of hypertension.

Subjects with signs of moderate to severe congestive heart failure greater than New York Heart Association class II, any significant valvular disease greater than grade II on a scale of I-IV, dilated or hypertrophic cardiomyopathy, congenital heart disease or a history of myocarditis were excluded from the study to avoid the role of disease-related atrial remodeling for the genesis of secondary AF. Further exclusion criteria were any severe concomitant pathologic condition such as hyperthyroidism, acute pneumonia or chronic lung disease, and neoplastic, renal and liver diseases. Subjects with palpitations without ECG documentation were excluded from both the case and control groups. Patients with pacemakers or cardioverter-defibrillator implantation before the occurrence of AF were also excluded. 

### Protocol

AF was defined as the replacement of sinus P waves by rapid oscillations or fibrillatory waves of varied in size, shape, and timing that were associated with an irregular ventricular response when atrioventricular conduction was intact. The presence of AF was determined from history, followed by serial ECG or ambulatory electrocardiographic monitoring. Lone AF was defined as AF occurring in individuals aged <65 years without hypertension, overt structural heart disease, or thyroid dysfunction, as determined by clinical examination, ECG, echocardiography, and thyroid function tests. Paroxysmal AF was defined as AF lasting >30 s that terminated spontaneously. The AF was classified as persistent when it lasted >7 days and required either pharmacological therapy or electrical cardioversion for termination. AF that was completely refractory to cardioversion or that was allowed to continue was classified as permanent [29]. Hypertension was defined according to the Seventh Report of the Joint National Committee on Prevention, Detection, Evaluation, and Treatment of High Blood Pressure criteria: systolic blood pressure equal to or over 140 mm Hg and/or diastolic blood pressure equal to or over 90 mm Hg (average of two measurements) or being on treatment with antihypertensive therapy [30]. Current smoking status was determined at the time of blood collection. Dyslipidemia was defined according to the Third Report of the National Cholesterol Education Program (NCEP) [31], and diabetes in agreement with the American Diabetes Association [32].

Twelve-lead ECG, routine laboratory screening, thyroid function test, and echocardiography were performed for all subjects. Transthoracic echocardiography was performed to measure the left atrial dimension (LAD) and left ventricular end-diastolic diameter (LVEDD); we also assessed the left ventricular ejection fraction (LVEF) and detected the presence of significant valvular disease, which was defined as moderate to severe valvular regurgitation or stenosis. 

### DNA Extraction and Genotyping

DNA was extracted from ethylene diamine tetraacetic acid anti-coagulated peripheral blood using a commercially available kit (Beijing CoWin Biotech Co., Ltd., Beijing, China) as described previously and stored at −20 °C [33].

The genotype of the -509C>T (rs1800469) polymorphism of the *TGFB1* gene was determined by polymerase chain reaction (PCR)-restriction fragment length polymorphism. In briefly, to detect the -509C>T polymorphism in the promoter, the primers 5`-CCC GGC TCC ATT TCC AGG TG-3` and 5`-GGT CAC CAG AGA AAG AGG AC-3` were used to PCR-amplify a fragment of the *TGFB1* gene [34]. The PCR was performed in a 25-μl reaction containing 25-50 ng genomic DNA, 1х PCR buffer, 2.0 mmol/l MgCl_2_, 200 μmol/l dNTP, 10 pmol of each primer, and 0.5 U of *Ex Taq* DNA polymerase (TaKaRa, Dalian, China). Amplification conditions were as follows: an initial activation step of 94°C for 5 min followed by 35 cycles of denaturation at 94°C for 30 s, annealing at 60°C for 30 s, extension at 72°C for 1 min, and a final extension step at 72°C for 10 min. 

The resultant 808-bp fragment was digested at 37°C for overnight using restriction enzyme *Eco*81I (TaKaRa, Darlian, China). *Eco*81I does not digest the T allele, but digest the C allele into 617-bp and 191-bp fragments.

Standard precautions to avoid contamination during PCR were taken. A negative control serum was included in each reaction to ensure specificity. Two researchers, who were blinded to the clinical data performed the genotyping independently. If there was any discordance between their findings, a third researcher would perform the genotyping and determine whether it was necessary to repeat the assay. Additionally, about 10% of the samples were randomly selected to perform the repeat assays, the results were 100% concordant. Furthermore, approximately 3% of the samples were randomly selected for direct sequencing, and the results were 100% concordant.

### Statistical Analysis

The Kolmogorov–Smirnov test was performed to evaluate the normality of data distribution. Continuous variables were expressed as mean ± SD or median and interquartile ranges as appropriate. Statistical significance of differences in quantitative variables was tested using the Student’s independent samples *t*-test and analysis of covariance or the nonparametric Wilcoxon test for normally or non-normally distributed variables, respectively. Categorical data are presented as frequencies (percentage). Differences between categorical variables, genotype/allele frequencies, and Hardy-Weinberg equilibrium were tested by the χ^2^ test or Fisher’s exact test. The association between the rs1800469 polymorphism and AF was assessed using logistic regression analysis under dominant, recessive, and additive genetic models. Variables such as age, sex, hypertension, and LAD were included in the multivariate model. We determined the odds ratio (OR) and 95% confidence interval. Analysis was performed using SPSS for Windows Version 11.5 (SPSS Inc., Chicago, IL, USA). Two-sided *P*-values <0.05 were considered significant. 

The software package Quanto 2.4 (http://hydra.usc.edu/gxe) was used for power calculations. Based on the minor allele frequency of 0.442 for rs1800469 as reported in the haplotype may of the CHB population, and assuming a dominant model, the study had >80% statistical power to detect an association (at *P* < 0.05) with an OR of 1.40, indicating a very low risk of a false-negative result.

## Results

There were 889 patients with AF and 1015 controls. Two hundred and seventy-eight cases of AF were lone AF (146 and 132 cases in the Southeast and Northeast Chinese population, respectively) and 334 cases of AF were diagnosed as paroxysmal AF (192 and 142 cases in the Southeast and Northeast Chinese population, respectively). As expected, AF patients in both study populations had larger LAD than the controls did (Southeast and Northeast Chinese cases vs. controls: 4.2 cm vs. 3.7 cm; and 4.1 cm vs. 3.6 cm, respectively, both *P* < 0.001). However, no significant differences were observed between the cases and controls with regard to age, sex distribution, and cardiovascular risk factors, such as hypertension, diabetes, dyslipidemia, and smoking habits in both study populations. Moreover, the distribution of other clinical characteristics such as height, weight and body mass index, LVEF and LVEDD were not significantly different in both the cases and controls (Table 1). 

**Table 1:** Clinical characteristics of cases and controls.

	Southeast Chinese population			Northeast Chinese population		
Characteristics	cases	Controls	*P*	cases	Controls	*P*
Sample size, n	581	723		308	292	
Age-range, years	21-92	35-93		32-90	40-92	
Age, years	70.6 ± 11.1	70.6 ± 10.2	0.992	66.4 ± 11.2	66.0 ± 10.1	0.671
Gender (male/female), n	361/220	444/279	0.789	212/96	197/95	0.720
Hypertension, n (%)	249 (42.9)	296 (41.0)	0.486	142 (46.1)	132 (45.2)	0.825
Diabetes mellitus, n (%)	100 (17.2)	106 (14.7)	0.209	65 (21.1)	69 (23.6)	0.458
Dyslipidemia, n (%)	110 (18.9)	151 (20.9)	0.381	70 (22.7)	59 (20.2)	0.452
Smoking, n (%)	130 (22.4)	187 (25.9)	0.144	109 (35.4)	92 (31.5)	0.314
Height,cm	165.5 ± 7.4	164.8 ± 7.2	0.103	168.1 ± 5.9	167.6 ± 5.5	0.242
Weight, kg	67.0 ± 9.5	66.2 ± 9.8	0.189	70.7 ± 8.0	70.4 ± 7.1	0.617
BMI, kg/m^2^	24.5 ± 2.7	24.3 ± 2.9	0.295	25.0 ± 2.4	25.1 ± 2.2	0.701
LAD, cm	4.2 (3.8-4.7)	3.7 (3.3-4.1)	< 0.001	4.1 (3.6-4.6)	3.6 (3.3-4.0)	< 0.001
LVEF, %	61 (58-64)	62 (59-64)	0.069	59 (56-64)	60 (57-64)	0.155
LVEDD, cm	4.7 (4.4-5.1)	4.7 (4.5-5.0)	0.332	4.7 (4.4-5.1)	4.7 (4.3-4.9)	0.216

Values are mean±SD, n (%), or median (interquartile range).

BMI indicates body mass index; LAD, left atrial dimension; LVEF, left ventricular ejection fraction; LVEDD, left ventricular end-diastolic diameter.

The demographic and clinical characteristics of the study populations are reported in Table S1. There were significant differences in the years of AF, age, height, weight, body mass index, and LVEF between the Southeast and Northeast Chinese population. Significant differences were also found in sex ratio, the ratio of paroxysmal, permanent, and lone AF, diabetes mellitus, smoking, and use of antiarrhythmic medication between the two populations. There were no significant differences in the ratio of hypertension, dyslipidemia, persistent AF, LAD, LVEDD, and use of anticoagulant and β-blocker medication between the two populations. 

The genotype distribution and allele frequencies of the rs1800469 polymorphism in the *TGFB1* gene are listed in Tables 2-4. The genotype distributions of the SNP in all groups were consistent with those expected for samples in Hardy-Weinberg equilibrium. Despite their geographical distance (Southeast and Northeast China), there was no difference in genotype distribution of the SNP between the two populations in control groups. The respective genotype and allele frequencies of the rs1800469 polymorphism were not different between AF patients and controls in the Southeast Chinese population, Northeast Chinese population and total study population. After adjustment for age, sex, hypertension, and LAD, there was no association between the SNP rs1800469 polymorphism and the risk of AF under the dominant, recessive and additive genetic models in the Southeast Chinese population, Northeast Chinese population and total study populations (Table 5). We further investigated whether the SNP was associated with lone AF and paroxysmal AF. There were no significant differences in the genotype and allele frequencies of the rs1800469 and no association between the rs1800469 and the risk of AF between lone AF patients and controls, and between paroxysmal AF patients and controls in the Southeast Chinese population, Northeast Chinese population and total study populations (Tables 2-5). 

**Table 2:** Genotype distribution and allele frequencies of -509C>T **polymorphism in the Southeast Chinese population.**

Genotypes and alleles	Controls (n = 723)	Cases (n = 581)	OR (95% CI)	*P*	Lone AF (n = 146)	OR (95% CI)	*P*	Paroxysmal AF (n = 192)	OR (95% CI)	*P*
CC	196 (27.1)	159 (27.4)	Reference		39 (26.7)	Reference		59 (30.8)	Reference	
CT	356 (49.2)	301 (51.8)	1.04 (0.80-1.35)	0.755	75 (51.4)	1.06 (0.69-1.62)	0.792	88 (45.8)	1.22 (0.84-1.77)	0.300
TT	171 (23.7)	121 (20.8)	0.87 (0.64-1.19)	0.392	32 (21.9)	0.94 (0.56-1.57)	0.814	45 (23.4)	1.14 (0.74-1.77)	0.548
HWE	0.706	0.328			0.719			0.277		
C	748 (51.7)	619 (53.3)	Reference		153 (52.4)	Reference		206 (53.6)	Reference	
T	698 (48.3)	543 (46.7)	0.94 (0.81-1.10)	0.433	139 (47.6)	0.97 (0.89-1.17)	0.835	178 (46.4)	1.08 (0.86-1.35)	0.504

Genotype distribution and allelic status were analyzed with the χ^2^ value test. Values are given as n (%).

Genotype distribution of the SNP between groups: for AF patients: global χ^2^=1.578, df=2, *P*=0.454; for lone AF patients: global χ^2^=0.275, df=2, *P*=0.872; for paroxysmal AF patients: global χ^2^=1.079, df=2, *P*=0.583;

AF, atrial fibrillation; HWE, *P*-value for exact Hardy–Weinberg equilibrium test; OR, odds ratio; CI, confidence interval.

**Table 3:** Genotype distribution and allele frequencies of -509C>T polymorphism in the Northeast Chinese population.

Genotypes and alleles	Controls (n = 292)	Cases (n = 308)	OR (95% CI)	*P*	Lone AF (n = 132)	OR (95% CI)	*P*	Paroxysmal AF (n = 142)	OR (95% CI)	*P*
CC	88 (30.1)	83 (26.9)	Reference		39 (29.6)	Reference		38 (26.8)	Reference	
CT	152 (52.1)	160 (52.0)	1.12 (0.77-1.62)	0.564	63 (47.7)	0.94 (0.58-1.51)	0.784	73 (51.4)	0.90 (0.56-1.44)	0.659
TT	52 (17.8)	65 (21.1)	1.33 (0.83-2.13)	0.242	30 (22.7)	1.30 (0.72-2.34)	0.378	31 (21.8)	0.72 (0.40-1.30)	0.279
HWE	0.329	0.456			0.638			0.715		
C	328 (56.2)	326 (52.9)	Reference		141 (53.4)	Reference		149 (52.5)	Reference	
T	256 (43.8)	290 (47.1)	1.14 (0.91-1.43)	0.260	123 (46.6)	1.12 (0.84-1.50)	0.455	135 (47.5)	0.86 (0.65-1.15)	0.304

Genotype distribution and allelic status were analyzed with the χ^2^ value test. Values are given as n (%).

Genotype distribution of the SNP between groups: for AF patients: global χ^2^=1.370, df=2, *P*=0.504; for lone AF patients: global χ^2^=1.484, df=2, *P*=0.476; for paroxysmal AF patients: global χ^2^=1.191, df=2, *P*=0.551;

AF, atrial fibrillation; HWE, P-value for exact Hardy–Weinberg equilibrium test; OR, odds ratio; CI, confidence interval.

**Table 4:** Genotype distribution and allele frequencies of -509C>T polymorphism in total study population.

Genotypes and alleles	Controls (n = 1015)	Cases (n = 889)	OR (95% CI)	*P*	Lone AF (n = 278)	OR (95% CI)	*P*	Paroxysmal AF (n = 334)	OR (95% CI)	*P*
CC	284 (27.9)	242 (27.2)	Reference		78 (28.1)	Reference		97 (29.0)	Reference	
CT	508 (50.1)	461 (51.9)	1.07 (0.86-1.32)	0.562	138 (49.6)	0.99 (0.72-1.35)	0.945	161 (48.2)	1.08 (0.81-1.44)	0.614
TT	223 (22.0)	186 (20.9)	0.98 (0.76-1.27)	0.872	62 (22.3)	1.01 (0.70-1.48)	0.949	76 (22.8)	1.00 (0.71-1.42)	0.990
HWE	0.883	0.219			0.948			0.557		
C	1076 (53.0)	945 (53.1)	Reference		294 (52.9)	Reference		355 (53.1)	Reference	
T	954 (47.0)	833 (46.9)	0.99 (0.88-1.13)	0.929	262 (47.1)	1.01 (0.83-1.21)	0.958	313 (46.9)	1.01 (0.84-1.20)	0.950

Genotype distribution and allelic status were analyzed with the χ^2^ value test. Values are given as n (%).

Genotype distribution of the SNP between groups: for AF patients: global χ^2^=0.645, df=2, *P*=0.724; for lone AF patients: global χ^2^=0.019, df=2, *P*=0.991; for paroxysmal AF patients: global χ^2^=0.343, df=2, *P*=0.843;

AF, atrial fibrillation; HWE, P-value for exact Hardy–Weinberg equilibrium test; OR, odds ratio; CI, confidence interval.

**Table 5:** Association between the -509C>T polymorphism with atrial fibrillation.

	Controls, n	Cases, n	M1 OR (95% CI)**^*a*^**	*P*	M2 OR (95% CI)**^*a*^**	*P*	M3 OR (95% CI)**^*a*^**	*P*
Southeast Chinese population	723	581	0.98 (0.73-1.30)	0.864	0.95 (0.73-1.24)	0.701	1.06 (0.84-1.34)	0.630
Northeast Chinese population	292	308	1.27 (0.82-1.97)	0.277	0.75 (0.51-1.10)	0.143	1.08 (0.77-1.53)	0.644
Total study population	1015	889	1.03 (0.81-1.30)	0.831	0.89 (0.72-1.11)	0.298	1.08 (0.89-1.31)	0.453
Lone AF	1015	278	1.00 (0.83-1.22)	0.964	0.98 (0.74-1.24)	0.737	1.05 (0.83-1.32)	0.690
Paroxysmal AF	1015	334	1.06 (0.90-1.24)	0.498	0.97 (0.73-1.30)	0.852	1.03 (0.85-1.24)	0.782

^a^ Adjusted for age, gender, hypertension and Left atrial dimension.

AF indicates atrial fibrillation; M1, dominant model; M2, recessive model; M3, additive model; OR, odds ratio; CI, confidence interval.

We investigated the association between the SNP genotype and other risk factors for AF. Table S2 lists the demographic data, clinical parameters, cardiovascular risk factors, and echocardiographic features of the three genotypes. None of these parameters was significantly different among the three groups.

## Discussion

To the best of our knowledge, this is the first study to exam the association between the rs1800469 polymorphism of the *TGFB1* gene and susceptibility to AF. We did not observe a significant difference in the genotype or allele frequencies of this SNP between AF patients and controls in the Southeast Chinese population, Northeast Chinese population and total study populations. Emerging data supports the premise that lone AF and paroxysmal AF are mechanistically different from non-lone and non-paroxymal AF phenotypes [35,36]. Thus, we performed a subanalysis of the AF population. There were similar findings for the lone AF and paroxysmal AF subgroups. Our results suggest that rs1800469 of the *TGFB1* gene is not related to AF susceptibility in the Chinese Han population. 

The *TGFB1* gene was analyzed as a candidate gene for AF because of its functional relation with the modulation of tissue fibrosis, upregulated expression in experimental and human AF [7–11,15,37], as well as selective atrial fibrosis and increased AF inducibility in cardiac-specific overexpression of constitutively active *TGFB1* in mice [12–14]. Promoter polymorphisms and non-synonymous variants are generally considered likely causal variants themselves and not merely markers associated by linkage disequilibrium to causal variants in their vicinity. Several such variants in the *TGFB1* gene with possible functional significance have been reported [16–23]. In the present study, we examined the association between rs1800469 of the *TGFB1* gene and susceptibility to AF. Cases and controls were matched by geographic regions, and the two study populations comprised a single Chinese Han ethnicity. There was no difference in genotype distribution of rs1800469 between controls in the two populations, rendering the risk of stratification of the populations for the polymorphism unlikely. The minor allele frequency of rs1800469 was 0.442, similar to that previously reported in normal Chinese Han populations [24–27,38]. Assuming an additive genetic model, our sample size had >80% power to detect an association with an OR for disease of >1.2. Under a dominant or a recessive model, association with an OR of >1.4 was detected. The power of the study was thus sufficient to detect associations of modest magnitude. We found no significant association between the common SNP rs1800469 of the *TGFB1* gene and the risk of AF. The rs1800473 polymorphism (868T>C, Leu10Pro), in strong linkage disequilibrium with the rs1800469 polymorphism in the Chinese Han population [24–26], is theoretically not related to susceptibility to AF. Our results were consistent with that of Wang et al. [39] who noted no significant association between the rs1800473 and the risk of AF in subjects with essential hypertension. These results indicated that the effect of the functional SNP effect did not significantly increase the risk of AF. Although we were unable to examine *TGFB1* gene expression, there is evidence that the SNP involved in the present candidate gene study has functional significance, and likely alters the promoter-reporter activity and plasma concentration of TGF-β1 [16,17]. However, the function of the TGF-β1 protein may be too complex for a single variant effect, and the true relationship between TGF-β1 and AF may lie in gene-gene or gene-environment interactions.

Recent genome-wide association studies of AF have focused on a few chromosomal regions with strong signals [40–43]. Despite such studies yielding promising results, a large percentage of the heritability of AF remains unexplained [44]. A growing number of researchers are turning to rare genetic changes with strong effects as important contributors [45,46]. The variants at positions -800, of codon 25 or 263 of the *TGFB1* gene are rare variants in unrelated Chinese Han individuals [24,26–28]. The rs1800468 polymorphism is located in a partial putative cyclic adenosine monophosphate response element-binding protein consensus site and may alter transcription [16]. The rs1800471 polymorphism was found to be related to TGF-β1 production in peripheral blood leukocytes [19–22], and functional analysis of rs1800472 with a luciferase reporter assay demonstrated that the protective variant I263 of the *TGFB1* gene is more active than the T263 variant [23]. These rare SNPs may be related with the risk of AF. However, our sample did not have sufficient power to detect the effect of any rare variants with a frequency of < 0.05. The rapid development of next-generation sequencing allows reliable detection of associations between rare variants and AF in larger populations [47,48]. Future research involving large-scale studies is needed to extend the present findings by detecting associations between these rare variants and the risk of AF in independent populations.

The present study has several limitations. First, different geographical and racial backgrounds of the individuals studied can affect the results of an association study. Therefore, our findings need to be confirmed in other populations. Moreover, potential selection bias might have occurred because control subjects selection in our study was hospital-based. Lastly, we cannot exclude the presence of asymptomatic AF in the controls even though we conducted an accurate interview weighted to symptoms related to dysrhythmias.

In summary, our results did not indicate a direct influence of the *TGFB1* rs1800469 functional gene polymorphism in increased risk of AF in two Chinese Han populations. Further studies are required to elucidate the role of this gene in the predisposition to AF.

## Supporting Information

Table S1 **Clinical Characteristics of the study population.**
(DOC)

Table S2 **Clinical features in study subjects by TGF-β1 -509C>T genotype.**
(DOC)
