# Supplementary material for: The TGFB1 Functional Polymorphism rs1800469 and Susceptibility to Atrial Fibrillation in Two Chinese Han Populations
Source: PLoS One. 2013 Dec 12;8(12):e83033. doi: 10.1371/journal.pone.0083033 (PMC3861462; doi:10.1371/journal.pone.0083033)
Supplement: Table S1 — Clinical Characteristics of the study population. (DOC) [file pone.0083033.s001.doc]

**Table S1 Clinical Characteristics of the study population**

| Characteristics | Southeast Chinese population | Northeast Chinese population | *P* |
| --- | --- | --- | --- |
| Sample size, n | 1304 | 600 |  |
| Age-range, years | 21-93 | 32-92 |  |
| Age, years | 70.6 ± 10.6 | 66.3 ± 10.7 | < 0.001 |
| Gender (male/female), n | 805/499 | 409/191 | 0.007 |
| Clinical classification of AF |  |  |  |
| Paroxysmal AF, n (%) | 192 (33.1) | 142 (46.1) | < 0.001 |
| Persistent AF, n (%) | 228 (39.2) | 108 (35.1) | 0.222 |
| Permanent AF, n (%) | 161 (27.7) | 58 (18.8) | 0.003 |
| Lone AF, n (%) | 146 (25.1) | 132 (42.9) | < 0.001 |
| Years since diagnosis of AF | 5.0 (2.0-9.25) | 4.0 (3.0-6.0) | < 0.001 |
| Hypertension, n (%) | 545 (41.8) | 274 (45.7) | 0.113 |
| Diabetes mellitus, n (%) | 206 (15.8) | 134 (22.3) | 0.001 |
| Dyslipidemia, n (%) | 261 (20.0) | 129 (21.5) | 0.456 |
| Smoking, n (%) | 317 (24.3) | 201 (33.5) | < 0.001 |
| Height, cm | 165 (159-170) | 168 (164-172) | < 0.001 |
| Weight, kg | 66.5 (60-72) | 70 (65-76) | < 0.001 |
| BMI, kg/m^2^ | 24.4 (22.5-26.1) | 25.1 (23.5-26.5) | < 0.001 |
| LAD, cm | 3.97 ± 0.65 | 3.91 ± 0.67 | 0.077 |
| LVEF, % | 62 (58-64) | 60 (57-64) | < 0.001 |
| LVEDD, cm | 4.75 ± 0.42 | 4.76 ± 0.57 | 0.719 |
| AF management |  |  |  |
| Oral anticoagulant, n (%) | 49 (8.4) | 38 (12.3) | 0.062 |
| Antiarrhythmic medication, n (%) | 106 (18.2) | 78 (25.3) | 0.013 |
| β-blocker, n (%) | 252 (43.4) | 117 (38.0) | 0.121 |

Values are mean±SD, n (%), or median (interquartile range).

AF indicates atrial fibrillation; BMI, body mass index; LAD, left atrial dimension; LVEF, left ventricular ejection fraction; LVEDD, left ventricular end-diastolic diameter.
