# Supplementary material for: The TGFB1 Functional Polymorphism rs1800469 and Susceptibility to Atrial Fibrillation in Two Chinese Han Populations
Source: PLoS One. 2013 Dec 12;8(12):e83033. doi: 10.1371/journal.pone.0083033 (PMC3861462; doi:10.1371/journal.pone.0083033)
Supplement: Table S2 — Clinical features in study subjects by TGF-β1 -509C>T genotype. (DOC) [file pone.0083033.s002.doc]

**Table S2 Clinical features in study subjects by TGF-β1 -509C>T genotype**

| Characteristics | CC | CT | TT | *P* |
| --- | --- | --- | --- | --- |
| Number of subjects, n | 526 | 969 | 409 |  |
| Age, years | 69.3 ± 11.2 | 69.4 ± 10.8 | 68.5 ± 10.3 | 0.371 |
| Gender (male/female), n | 348/178 | 594/375 | 248/161 | 0.122 |
| Hypertension, n (%) | 245 (46.6) | 405 (41.8) | 162 (39.6) | 0.076 |
| Diabetes mellitus, n (%) | 128 (23.8) | 206 (21.3) | 86 (21.0) | 0.333 |
| Smoking, n (%) | 154 (29.3) | 260 (26.8) | 104 (25.4) | 0.394 |
| Height, cm | 166.4 ± 7.0 | 166.0 ± 6.9 | 165.4 ± 7.0 | 0.089 |
| Weight, kg | 68.6 ± 9.5 | 67.7 ± 9.2 | 67.2 ± 9.0 | 0.074 |
| BMI, kg/m^2^ | 24.7 ± 2.7 | 24.5 ± 2.7 | 24.6 ± 2.7 | 0.416 |
| LAD, cm | 3.99 ± 0.69 | 3.96 ± 0.65 | 3.89 ± 0.63 | 0.066 |
| LVEF, % | 60.9 ± 4.5 | 61.2 ± 4.6 | 61.1 ± 4.1 | 0.213 |
| LVEDD, cm | 4.75 ± 0.51 | 4.76 ± 0.46 | 4.71 ± 0.44 | 0.187 |

Values are mean±SD or n (%)

BMI indicates body mass index; LAD, left atrial dimension; LVEF, left ventricular ejection fraction; LVEDD, left ventricular end-diastolic diameter.
